# Effects of Physicochemical Parameters on Struvite Crystallization Based on Kinetics

**DOI:** 10.3390/ijerph19127204

**Published:** 2022-06-12

**Authors:** Jinzhu Wu, Yifan Li, Baojian Xu, Mei Li, Jing Wang, Yuanyuan Shao, Feiyong Chen, Meng Sun, Bing Liu

**Affiliations:** 1 Resources and Environment Innovation Institute, School of Municipal and Environmental Engineering, Shandong Jianzhu University, Jinan 250101, China; wjz408858154@126.com (J.W.); yifan_li2019@163.com (Y.L.); x17865178852@163.com (B.X.); limei@sdjzu.edu.cn (M.L.); jwang@sdjzu.edu.cn (J.W.); shaoyuanyuan@sdjzu.edu.cn (Y.S.); ctokyo@hotmail.com (F.C.); 2 Faculty of Environmental Engineering, The University of Kitakyushu, 1-1, Hibikino, Wakamatsu, Kitakyushu 802-8577, Japan; m-sun@kitakyu-u.ac.jp

**Keywords:** phosphate recovery, struvite, chemical precipitation, crystallization kinetics

## Abstract

The precipitation of struvite (MgNH_4_PO_4_·6H_2_O) is considered to be a promising method for the recovery of phosphate from wastewater. In this review, the kinetic models, which are commonly used to explain the process of struvite crystallization, are described. The mixed-suspension mixed-product removal (MSMPR) model is based on the population balance equation (the size-dependent growth model and the size-independent growth model). Thereafter, the first-order kinetic fitting model that aligned with concentration changes in the substrate is summarized. Finally, the several physical and chemical factors that affected the efficiency of struvite crystallization are determined. The supersaturation ratio, which is seen as the driving force of struvite crystallization, is the main factor that influences crystallization; however, it cannot be used in practical applications of engineering because it is indirectly associated with the following factors: pH, the molar ratio of Mg:N:P, and the interference of foreign impurities. In this study, we present conclusions that should be used to guide further research studies, and encourage the engineering practice of wastewater treatment with struvite precipitation.

## 1. Introduction

Phosphorus is a limiting nutrient in organisms of an ecosystem. Whenever there is an excessive discharge of phosphorus in the ecosystem, it leads to eutrophication of water and causes environmental damage [1]. Although the reserves of phosphorus are limited and nonrenewable, the consumption of phosphorus has increased steadily in recent decades [2,3]. Therefore, it is essential to recycle the resources of phosphorus and to gain social and economic benefits [4]. The recovery of phosphate is achieved by crystallizing struvite (MgNH_4_PO_4_·6H_2_O), which is an important phosphate mineral found in wastewater [5]. The recovery of phosphate is possible from this mineral because the phosphate removal rate is excellent in the crystallization process. Furthermore, the isolated phosphate can be fixed as a fertilizer, thereby enhancing its economic value [6,7]. Traditional phosphorus fertilizers are mostly water soluble. Phosphorus in farmland enters rivers with precipitation and drainage, resulting in eutrophication of a water body and lack of phosphorus in the later stage of crop growth. Different from traditional fertilizers, struvite has low solubility in water and is not prone to leaching loss [8]. It is a slow-release phosphate fertilizer. Struvite was first found in a sludge digestion system [9,10]. It is a crystal formed by chelating equimolar concentrations of magnesium, ammonium, and phosphate with six water molecules [11]. The crystallization process can be completed in less than one minute. The crystallization of struvite is favored as its solubility is low in water. The solubility product constant of struvite (Ksp) is 13.26 in water [12]. 

The crystallization of struvite is a complex process, which is affected by the following physicochemical parameters: pH, the molar ratio of Mg:N:P, temperature, and foreign impurities [13,14]. Therefore, the various physicochemical parameters that influence the process of struvite crystallization need to be understood. In many current studies, phosphorus removal rate has been used to characterize the effects of various physicochemical parameters on struvite crystallization. However, the key to phosphorus recovery not only needs high phosphorus removal rate, but also needs to form high-quality struvite. Therefore, the model of struvite crystallization needs to be established to investigate the effect of physicochemical parameters on the nucleation and growth of struvite from the perspective of kinetics. With this knowledge, highly pure struvite was synthesized, which can be further used for highly efficient phosphate recovery [15]. In addition, struvite crystallization is favored by establishing the proper equilibrium and growth rate of crystals. Thus, the uncertainty can be reduced in the process, design, and operation of crystallization units [16].

Currently, the dynamic kinetics of struvite crystallization are described by the following models: the population density model [17], the surface growth model [18], and the first-order dynamic model of substrate concentration [19]. Furthermore, the population density model describes how the total rate of change in the crystal number occurs due to variations in following parameters: diameter, surface area, volume, shape, etc.

The objective of this study is to summarize the common kinetic models used in struvite crystallization. Thereafter, the effects of various physical and chemical parameters on the crystallization efficiency of struvite are discussed. Subsequently, the results were used to understand the perspective of crystallization kinetics, which can be further used as a reference work in the development of an efficient technology for phosphate recovery.

## 2. Model of Struvite Crystallization

In wastewater, supersaturated magnesium ions (${\mathrm{Mg}}^{2+}$), ammonium (${\mathrm{NH}}_{4}^{+}$), and orthophosphate (${\mathrm{PO}}_{4}^{3-}$, ${\mathrm{HPO}}_{4}^{2-}$ and $H_{2}{\mathrm{PO}}_{4}^{-}$) combine to form struvite (MgNH_4_PO_4_·6H_2_O). Struvite is formed through the following equation [20]:

(1) $${\mathrm{Mg}}^{2+}{+\mathrm{NH}}_{4}^{+}{+\mathrm{PO}}_{4}^{3-}{+6H}_{2}O\to {\mathrm{MgNH}}_{4}{\mathrm{PO}}_{4}{\cdot 6H}_{2}O$$

The driving force (F) of struvite crystallization is defined as follows [21]:

(2) $$F=-\frac{R_{g}T}{\alpha }\ln\Omega$$

where $R_{g}$ is the gas constant (8.31 J·K^−1^·mol^−1^); α is 3; T is the absolute temperature; and Ω is the supersaturation ratio of the solution, which is defined as the ratio of the ion activity product (IAP) in the solution to the solubility product (K_sp_) of the struvite crystal.

(3) $$\Omega =\frac{\left({\mathrm{Mg}}^{2+}\right)\left({\mathrm{NH}}_{4}^{+}\right){(\mathrm{PO}}_{4}^{3-})}{K_{\mathrm{sp}}}$$

where $\left({\mathrm{Mg}}^{2+}\right)\;\left({\mathrm{NH}}_{4}^{+}\right){\;(\mathrm{PO}}_{4}^{3-})$ denotes the activity of ${\mathrm{Mg}}^{2+}$, ${\mathrm{NH}}_{4}^{+}$, and ${\mathrm{PO}}_{4}^{3-}$ ions in the solution, and it is calculated by using the Davies model; and K_sp_ is the solubility product of struvite crystal.

The crystallization process of struvite is classified into two primary stages: the nucleation and the growth of crystals. In the nucleation phase, the ions combine with each other to form a crystalline embryo. The growth of crystals ends when an equilibrium is reached in the reaction mixture [14]. In general, nucleation is classified into two types: homogenous nucleation is a spontaneous process; heterogeneous nucleation is brought about by including suspended solid impurities in the solution [22]. According to classical nucleation theory, the homogenous nucleation rate J can be calculated as follows [23]:

(4) $$J=\delta \exp\left(-\frac{{\beta \gamma }^{3}v^{2}}{{\left(\mathrm{kT}\right)}^{3}{\left(\ln\Omega \right)}^{2}}\right)$$

where β is a shape factor; v is the molecular volume; γ is the surface energy of the crystal; k is the Boltzmann’s constant (1.381 × 10^−23^ J/K); and δ is the pre-exponential factor [24].

(5) $$\delta =\left(\frac{D}{d^{5}N}\right){\left(\frac{4\Delta G}{3\pi \mathrm{kT}}\right)}^{1/2}$$

where D is the diffusion coefficient; d is the interplanar distance of the crystalline lattice; N is the number of molecules that form a critical sized nucleus; and ΔG is the change in Gibb’s free energy, which occurs due to the formation of a critical nucleus.

The induction time of a crystal is defined as the time expended from the supersaturation of the solution to the solid formation; it can be determined from the changes caused by crystallization in solution properties such as pH and conductivity [18]. By assuming that the induction time is much lesser than the nucleation time, the induction time (τ) was determined as follows:

(6) $$\tau =\frac{A}{{\left(\log\Omega \right)}^{2}}-B$$

where A = ${\beta \gamma }^{3}v^{2}/(2.303{\mathrm{kT})}^{3}$ and B = logξ.

The growth rate of struvite crystals is calculated by using the following equation:

(7) $${R=k}_{r}{{(\Omega }^{\frac{1}{3}}-1)}^{n}$$

where $k_{r}$ is the reaction coefficient; and n is the apparent reaction order. 

In previous studies, researchers have determined the kinetics of struvite crystallization by using the mixed-suspension mixed-product removal (MSMPR) model. This model is based on the population balance equation. It is a method used to estimate the kinetic parameters of the crystallization of struvite, which depend on the crystal size distribution (CSD) of the product [17,25,26]. The crystal growth rate and nucleation rate affect CSD in solution, which is the key to control the quality of struvite [27]. The MSMPR model includes the size-dependent growth (SDG) model and the simplified size-independent growth model (SID).

In the MSMPR model, the population density of the mean size of struvite crystal $n_{i}\left(L_{i}\right)\;\mathrm{is}\;$based on CSD data. The model is described by the following equation [17]:

(8) $$n_{i}\left(L_{i}\right)=\frac{m_{i}\left(L_{i}\right)}{k_{v}{\rho L}_{i}^{3}\Delta L_{i}V_{w}}=\frac{V_{i}\left(L_{i}\right)}{k_{v}L_{i}^{3}\Delta L_{i}V_{w}}$$

where $L_{i}$ denotes the mean size of the ith crystal; $m_{i}$ is the mass of the crystal; $V_{i}$ is the volume of the crystal; $k_{v}$ is the shape factor; ρ is the density of the crystal; $\Delta L_{i}$ denotes the crystal size range; $\mathrm{and}\;V_{w}$ is the total volume of the system.

Using the assumption of the SID model, we calculated the linear growth rate G of the crystal by the following equation:

(9) $$\mathrm{lnn}\left(L\right)=-\frac{L}{\mathrm{tG}}{+\mathrm{lnn}}_{0}$$

where t is the mean retention time of the crystal; lnn(L) has a linear relationship with L, and its slope is 1 /tG. After determining the value of t, G is obtained by fitting the slope of the straight line. The intercept of the fitting line is denoted as ${\mathrm{lnn}}_{0}$. The nucleation rate (B) can be written according to the following equation [25]:

(10) $${B=n}_{0}G$$

Since the crystals are of different sizes, their growth rates are also different [28]. Therefore, the SDG model is introduced, and its parameters are defined in Table 1:

The SIG model is a simplification of the SDG model, which is only used to process the growth data of smaller crystals, but it should be noted that the simulation of G(SIG) and G_m_ (SDG) by the two models is similar [33].

The first-order kinetic model is used to assess the growth rate of struvite crystallization, which can be further described as follows [19,34]:

(11) $$\;\ln\left(C-C_{\mathrm{eq}}\right)=-\mathrm{kt}+\ln(C-C_{0})$$

where C is the concentration of the substrate at time t; $C_{\mathrm{eq}}$ is the equilibrium concentration of the substrate; $C_{0}$ is initial concentration of substrate; and k is the rate constant of first-order reaction. The parameter values in some studies are summarized in Table 2.

It should be noted that most kinetic studies regard the crystallization of struvite as a first-order irreversible reaction. In fact, the crystallization behavior of struvite is reversible [39].

In addition, the reaction order method [40] and the chemical potential gradient model [41] were used to simulate the rate of struvite crystallization, providing new insights into the crystallization behavior of struvite.

## 3. The Parameters That Influence Struvite Crystallization

Supersaturation is a crucial factor that affects the rate of struvite crystallization. However, it should be emphasized that supersaturation is indirectly affected by the following factors: pH, concentration of the solute, temperature, and the presence of foreign impurities in the solution. Due to these factors, supersaturated crystals of struvite cannot be used in practical applications of engineering. In this section, how the aforementioned factors affect the formation of struvite crystals is summarized.

### 3.1. pH

The dissolution and crystallization of struvite occur simultaneously in an aqueous solution [42]. Initially, the solubility of struvite decreases, but it increases when the pH value increases subsequently [43]. The solubility of struvite is minimum when the pH value is maintained between 8.5 and 9.0 [44]. In acidic conditions, struvite decomposes to form an amorphous crystal. At this stage, the concentration of $H_{2}{\mathrm{PO}}_{4}^{-}$ ions decreases [45]. Furthermore, when the pH is increased above the value of 9, the solubility of struvite starts increasing. This happens when ionized ammonium ion (${\mathrm{NH}}_{4}^{+}$) is converted into an unionized state of ammonia (NH_3_), causing a decrease in supersaturation [46]. 

In solutions with different pH, the ion concentration profiles of orthophosphate and ammonia are different. This implies that the supersaturation ratio of the solution is affected by pH, causing changes in the growth rates of struvite crystals. When the pH value of the solution is 8.0, 8.5, and 9.0, the kinetics of struvite crystals growth follows a first-order equation. Its first-order rate constants are 3.7, 5.1, and 6.9 h^−1^ at pH values of 8.0, 8.5, and 9.0, respectively [47]. Moreover, the first-order rate constant of struvite crystals growth increases when the pH value is increased from 8.4 to 9.0 [37].

The zeta potential is affected by the interaction between particles in an aqueous solution. A change in pH of the solution can change the value of the zeta potential, thereby affecting the growth kinetics of the struvite crystal. When the pH value is increased steadily, the zeta potential becomes more negative. Consequently, the growth rate of crystals is accelerated in the solution [23,48]. However, if the initial value of pH is high, the positive zeta potential is high, and the nucleation time is increased substantially [24].

The pH value is varied to modify the shape and the linear growth rate of struvite crystals. By increasing the pH value, the size and the thickness of struvite crystal are decreased. Matynia, et al. [49] reported that when the pH value of the solution increased from 8 to 10, the average crystal size of struvite decreased by 5.5 times. Similarly, in the study of Anna, et al. [50], the average crystal size of struvite decreased by 3 times when the pH value of the solution increased from 9 to 11. Mazienczuk, et al. [25] reported that when the range of pH values was controlled within 9–11, the relationship between crystal size, linear growth rate, and pH value was as follows:

(12) $${\;L}_{m}=2.86{\times 10}^{5}{\mathrm{pH}}^{-5.44}t^{0.328}{\;R}^{2}=0.808$$

(13) $$\;G=5.39{\times 10}^{-4}{\mathrm{pH}}^{-2.99}t^{0.683}{\;R}^{2}=0.905$$

In addition, Mg^2+^ ions form crystals of Mg (OH)_2_ salt at higher pH values. This supplementary crystallization process interferes with the formation of struvite crystals. Primarily, if the pH value is greater than 10.5, a Mg_3_(PO_4_)_2_ compound is formed, and it is insoluble in a strongly alkaline environment [51]. The crystallization of Mg_3_(PO_4_)_2_ and Mg(OH)_2_ is described by the following equations [52]:

(14) $${\;3\mathrm{Mg}}^{2+}{+2\mathrm{PO}}_{4}^{3-}\to {\mathrm{Mg}}_{3}{\left({\mathrm{PO}}_{4}\right)}_{2}$$

(15) $${\mathrm{Mg}}^{2+}{+2H}_{2}O↔\mathrm{Mg}{\left(\mathrm{OH}\right)}_{2}↓{+2H}^{+}$$

In summary, the effect of pH on struvite crystallization is summarized as follows: Firstly, the pH value affects the formation of new ion species in the solution, which encourages the formation of other crystals. Secondly, it affects the solubility and zeta potential of struvite crystals. When the pH value of the solution is less than 7, there is formation of mainly orthophosphate $H_{2}{\mathrm{PO}}_{4}^{-}$. At this acidic pH value, the crystallization and the growth of struvite are significantly inhibited. However, at a high pH value (≥11), other precipitates are formed and ammonia becomes volatized. These events lead to the dissolution of struvite, and the nucleation and crystallization of struvite are inhibited. In addition, as the pH value of solution increases, the nucleation rate increases in struvite crystals and the growth rate of struvite crystals decreases, which produces a large number of small-sized struvite.

### 3.2. Molar Ratio of Mg:N:P

As shown in Equation (1), the theoretical molar ratio of Mg:P:N is 1:1:1. However, to ensure that phosphorus is removed with high efficiency, an excessive amount of ammonium or magnesium or both elements is often added. In a study conducted by Gong, et al. [53], the molar ratio of Mg:P was increased from 0.8 to 1.2. Consequently, the rate of phosphorus removal increased from 80.8 to 95.5%. This is because other insoluble compounds of magnesium and phosphorus were formed along with struvite crystals [54]. Similarly, Hutnik et al. [55] reported that the addition of excess magnesium ion is not only conducive to the formation of larger struvite crystals, but also can remove a variety of impurities in wastewater. Furthermore, at a given value of pH, an excessive concentration of phosphorus was maintained. In these conditions, the degree of supersaturation depended only on the concentration of magnesium and ammonium ions [56]. The yield of struvite increased when the concentration of Mg^2+^ and NH_4_^+^ was increased. This indicates that the saturation of struvite is directly proportional to the logarithm of the ionic concentrations in the crystal [57].

The molar ratio of Mg:N:P elements affect the supersaturation, which further impacts the crystalline morphology of struvite. When the relative supersaturation  ratio σ ($\Omega^{1/3}$ − 1) is within the range of 1.0–1.5, the struvite crystal develops into the shape of a coffin. When the relative supersaturation ratio is within the range of 1.5–3.0, the struvite crystal possesses a twin/polycrystalline state and develops into an X-shaped and needle-like shaped crystal [58]. In addition, the kinetic parameters of struvite crystallization are affected by supersaturation. Galbraith et al. [59] reported that struvite crystals more likely aggregate with supersaturation increases. Similarly, Koralewska et al. [60] reported that when Mg^2+^ increased from 0.1 to 1 mass-%; Gm and G_0_ of struvite crystal increased from 8.38 × 10^−9^, 1.48 × 10^−10^ to 1.18 × 10^−8^, 6.62 × 10^−10^ m/s; and B from 6.22 × 10^12^ to 4.67 × 10^14^/m^3^·s. Higher supersaturation is beneficial to the nucleation and growth of struvite, and leads to a faster precipitation process and greater particle density.

### 3.3. The Effects of Foreign Impurities on Struvite Crystallization

It is established that Ca^2+^ or CO_3_^2−^ ions extend the induction time of crystallization and inhibit the growth rate of crystals. When calcium ions are absorbed on the surface of a crystal, the binding site of ammonia is occupied. Consequently, the crystallization of struvite is inhibited [61]. Furthermore, calcium ions can consume phosphate and then influence an oversaturated form of struvite. Calcium ions interact with phosphate or carbonate ions to form calcium phosphate (usually hydroxyapatite) or calcium carbonate (usually calcite), respectively. Please refer to Equations (16) and (17) [62]:

(16) $${5\mathrm{Ca}}^{2+}{+3\mathrm{PO}}_{4}^{3-}{+H}_{2}O\to {\mathrm{Ca}}_{5}{\left({\mathrm{PO}}_{4}\right)}_{3}{\mathrm{OH}+H}^{+}$$

(17) $${\mathrm{Mg}}^{2+}{+2H}_{2}O↔\mathrm{Mg}{\left(\mathrm{OH}\right)}_{2}↓{+2H}^{+}$$

**Figure 1:**
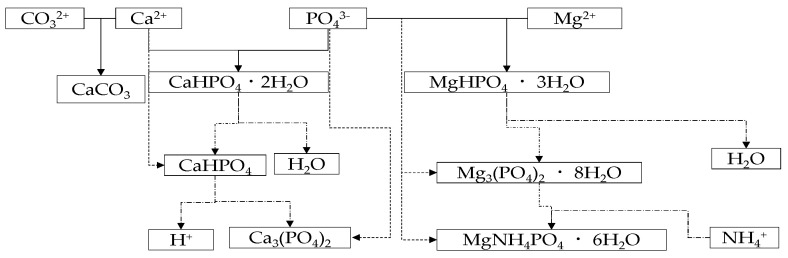
Reaction map of calcium and magnesium with soluble phosphate (−: primary solid crystallization; − · −: transform to a secondary solid; − − −: precipitate of the secondary solid).

Yaakoubi et al. [63] reported that the reaction processes of struvite crystallization were dramatically improved when Ca^2+^ or Mg^2+^ ions were added into an aqueous solution containing phosphate and ammonia species. As shown in Figure 1, precipitates of CaHPO_4_·2H_2_O and MgHPO_4_·3H_2_O were obtained initially due to high solubility and weak thermodynamics. Subsequently, a more stable form of the species was generated as CaHPO_4_ and Mg_3_(PO_4_)_2_·8H_2_O. In the same manner, Ca_3_(PO_4_)_2_ and Mg (NH_4_) PO_4_·6H_2_O were eventually achieved.

In a study conducted by Hutnik et al. [26], the SDG model was used to determine how calcium ions affect the crystallization of struvite. When the concentration of calcium ions was increased from 100 mg/L to 2000 mg/L, the nuclear growth rate decreased significantly from 2.30 × 10^−11^ to 2.09 × 10^−12^ m/s. Moreover, the linear growth rate of crystals also decreased significantly from 1.71 × 10^−8^ to 9.10 × 10^−9^ m/s. Nevertheless, the nucleation rate of struvite increased 160 times. In contrast, the purity of struvite decreased by 8.1 mass%. Moreover, the process of struvite crystallization slowed down when the molar ratio of Ca^2+^/Mg^2+^ ions became greater than 0.2. The formation of struvite crystals was significantly inhibited, and the purity decreased when the molar ratio was increased from 0.5 to 1.0. Moreover, the formation and the purity of struvite crystals declined when the molar ratio of Ca^2+^/PO_4_^3−^ was increased from 0.5 to 1.0 [20].

The nucleation and growth of struvite crystals improve with the presence of copper ions in the solution. This is because copper ions increase the linear growth rate of struvite crystals. In contrast, they also cause a decline in the nucleation rate, thereby facilitating the formation of large-sized crystals [64]. A similar phenomenon was observed when the solution was treated with aluminum ions. When the concentration of aluminum ions was increased from 10 to 100 mg/L, the nucleation rate of struvite decreased by 5%. In contrast, the linear growth rate of struvite crystals increased by 8%, and the average size of crystals increased by 22% [17]. In addition, Hutnik et al. [65] reported that the existence of K^+^ increases the growth rate of struvite crystal, and the average size of struvite was about 46 um.

Magnesium chloride is often used as a source of magnesium and an additive. Ariyanto, et al. [14] determined how chloride ions affect the crystallization efficiency of struvite. The results indicate that excessive chloride ions increase the activation energy of struvite nucleation. Moreover, they also increase the induction time of struvite. Ping, et al. [66] reported that the efficiency of phosphorus removal increased when the total suspended solids were present at higher concentrations in actual wastewater, and the diameter and purity of struvite crystals decreased significantly.

**Figure 2:**
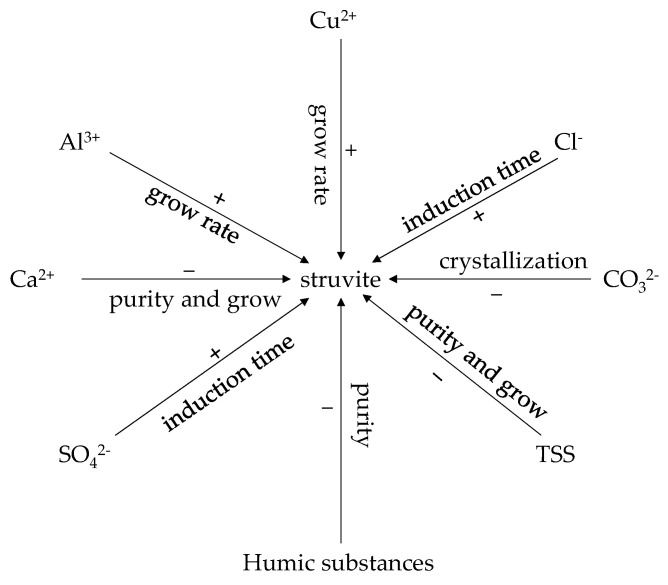
The influence of foreign impurities on struvite: +: positive effect, −: negative effect.

The presence of humic acid in wastewater clearly restricted the removal of phosphate, and the removal efficiency dropped from 97.47% to 80.80% [67]. This is because humic acid contains several carboxylic groups (-COOH). Therefore, humic acid shows a higher affinity towards Mg^2+^ and NH_4_^+^ ions, facilitating the formation of the following complexes: [C_3_H_5_O(COO)_3_]_2_Mg_3_ and C_3_H_5_O(COO)_3_(NH_4_)_3_ [68]. In addition, Qi Zhang, et al. [69] indicated that the crystallization of struvite was inhibited by the presence of impurities, which were formed when the dissolved humic acid combined with the seed crystal. Moreover, humic acid also endorsed a simple coverage of the available sites on the crystal nucleus, which slowed down the crystallization rate of struvite. The morphology of the collected struvite crystals changed from a prismatic to pyramidal shape because co-crystallization occurred due to the presence of humic acid on the surface of crystals [70]. This point of view is also supported by Wei, et al. [21], who reported that the change in the morphology of crystals was most likely triggered by the following functional groups: amides, humic acid-Mg^2+^ complex, and phosphate ester. These chemical moieties were formed due to the interaction between humic acid and struvite crystals. As shown in Figure 2, foreign impurities have a significant influence on the formation of struvite crystals.

In summary, the effect of foreign impurities on struvite crystallization is complex, and its mechanism needs to be further studied. However, it can be noted that metal cations lead to the decrease in the nucleation rate and the increase in the growth rate of struvite.

## 4. Conclusions

In this study, the kinetic model of struvite crystallization was explained. The MSMPR model, which is based on a statistical method, can be used to accurately predict the nucleation and crystallization of struvite; however, it cannot describe the mechanism of crystallization. The kinetic model of struvite crystallization needs to be further elucidated.

The lower the nucleation rate, the higher the linear growth rate of crystals; these conditions are considered to be ideal for struvite crystallization. Moreover, the supersaturation rate also needs to be controlled in this process. The supersaturation ratio of the solution is affected by the following parameters: pH, the molar ratio of Mg:N:P elements, and the interference of external ions. All these factors also affect the nucleation and growth rate of struvite crystals.

## Figures and Tables

**Table 1:** The SDG models.

Linear Growth Rate G	Mean Size Population Density n (L)	References
${G=G}_{0}{(1+\frac{L}{{\mathrm{tG}}_{0}})}^{b}$ b < 1	$n\left(L\right){=n}_{0}{\left(1+\frac{L}{{\mathrm{tG}}_{0}}\right)}^{-b}\exp\left[\frac{1}{1-b}-\frac{{\left(1+\frac{L}{{\mathrm{tG}}_{0}}\right)}^{-b}}{1-b}\right]$	[29]
${G=G}_{m}\left(1-e^{-a(L+c)}\right)$ a > 0, c ≠ 0	$n\left(L\right){=n}_{0}e^{\mathrm{aL}}{\left(\frac{e^{a(L+c)}-1}{e^{\mathrm{ac}}-1}\right)}^{(-1-{\mathrm{atG}}_{m}{)/\mathrm{atG}}_{m}}$	[30]
${G=G}_{m}\frac{e^{\mathrm{aL}}-b}{e^{\mathrm{aL}}-c}$	$n\left(L\right){=n}_{0}\left(\frac{e^{\mathrm{aL}}-c}{1-c}\right){\left(\frac{e^{\mathrm{aL}}-b}{1-b}\right)}^{\frac{c-a-{\mathrm{abtG}}_{m}}{{\mathrm{abtG}}_{m}}e^{-\frac{\mathrm{cL}}{{\mathrm{btG}}_{m}}}}$	[31]
${G=G}_{m}-{(G}_{m}-G_{0}{)e}^{\mathrm{aL}}$	$n\left(L\right){=n}_{0}e^{\frac{L}{{\mathrm{tG}}_{m}}}{\left(\frac{G_{0}}{G_{m}-{(G}_{m}-G_{0}{)e}^{\mathrm{aL}}}\right)}^{\frac{1}{{\mathrm{atG}}_{m}}+1}$	[32]

where $G_{0}$ represents the linear growth rate of nuclei, which further grow into zero-size crystals; $G_{m}$ represents the limiting linear growth rate of most giant crystals; a, b, and c represent the empirical constants.

**Table 2:** An overview of previous kinetics studies of struvite crystallization.

Research Object	pH (−)	Temperature ℃	Molar Ratio (Mg:p)	k (min^−1^)	R^2^ (−)	References
Phosphate concentration	7.5	22–25	1.5	0.039	>0.92	[35]
Phosphate concentration	8.51	20	1.6	0.045	0.97	[36]
Phosphate concentration	8.4	22–24	1.2	0.061	0.96	[37]
Magnesium concentration	9.0	30	1.0	0.109	0.99	[19]
Magnesium concentration	9.0	20	0.5	0.156	>0.92	[38]

## Data Availability

Not applicable.
